# A Genetic Transformation Method for Cadmium Hyperaccumulator *Sedum plumbizincicola* and Non-hyperaccumulating Ecotype of *Sedum alfredii*

**DOI:** 10.3389/fpls.2017.01047

**Published:** 2017-06-16

**Authors:** Huan Liu, Haixia Zhao, Longhua Wu, Wenzhong Xu

**Affiliations:** ^1^Key Laboratory of Plant Resources, Institute of Botany, Chinese Academy of SciencesBeijing, China; ^2^University of Chinese Academy of SciencesBeijing, China; ^3^Key Laboratory of Soil Environment and Pollution Remediation, Institute of Soil Science, Chinese Academy of SciencesNanjing, China

**Keywords:** *Agrobacterium tumefaciens*, genetic transformation, cadmium, hyperaccumulator, multiple shoot buds, *Sedum alfredii*, *Sedum plumbizincicola*

## Abstract

The present study demonstrates the development of an *Agrobacterium*-mediated genetic transformation method for species of the *Sedum* genus, which includes the Cd/Zn hyperaccumulator *Sedum plumbizincicola* and the non-hyperaccumulating ecotype of *S. alfredii*. Multiple shoots were induced from stem nodes of two *Sedum* plants using Murashige and Skoog (MS) medium containing 0.1 mg/L cytokinin 6-benzyladenine (6-BA) and 1.0 mg/L auxin 1-naphthaleneacetic acid (NAA). The shoot primordia were used as direct targets for *Agrobacterium* infection. Selection on hygromycin was highly effective in generating *Agrobacterium*-transformed explants. This callus-free procedure allowed us to obtain transgenic plantlets after rooting hygromycin-resistant shoots on phytohormone-free MS medium containing the antibiotic. The presence and expression of the reporter genes *gusA* and *GFP* in transgenic plants were confirmed by a real-time polymerase chain reaction, histochemical GUS assays, and confocal microscopy. This reliable method for genetic transformation of *Sedum* plants will help us to understand gene functions and the molecular mechanisms underlying Cd hypertolerance and hyperaccumulation in these species.

## Introduction

The genus *Sedum* L. is the most species-rich member of the family Crassulaceae (Nikulina et al., 2016). There are about 420 species (Thiede and Eggli, 2007), most of which can endure harsh environments ranging from very cold to hot temperatures and dry conditions. In response to conditions of drought stress, many *Sedum* species evolve crassulacean acid metabolism (CAM) system to take up inorganic carbon in the night (Castillo, 1996; Smirnoff, 1996; Winter and Holtum, 2014). Sedums are also usually cultivated as ornamental plants due to their interesting appearance, diversified phenotypes and extensive adaptability. Some sedums, such as *Sedum sarmentosum, S. lineare*, and *S. emarginatum*, are important medical plants used to treat hepatitis, dysentery, herpes zoster and swelling (Wang et al., 2014). Recently, *S. plumbizincicola* and population of *S. alfredii*, native to cadmium (Cd)/zinc (Zn) mining areas in Southeast China, were remarkable Zn and Cd hyperaccumulators that have high capacity to accumulate, translocate, and tolerate high concentrations of heavy metals (Yang et al., 2004; Wu et al., 2013).

The rare plant species identified as Cd hyperaccumulators are able to accumulate Cd to 100 ppm or 0.01% in the dry shoot biomass when growing in their natural habitats (Clemens, 2001; van der Ent et al., 2013). It may be possible in the future to use these plants, or biotechnology based on an understanding of the relevant molecular mechanisms, for phytoremediation of Cd contaminated environments, to decrease soil toxicity, and/or to enhance crop production (Verbruggen et al., 2009; Hanikenne and Nouet, 2011; Dalcorso et al., 2013; Verbruggen et al., 2013). *S. plumbizincicola* growing in field soils is able to accumulate high concentrations of Cd and Zn in shoots (>400 mg Cd or 10 000 mg Zn kg^-1^ dry weight, respectively) and has a shoot/root concentration ratio > 1 (Deng et al., 2007; Liu et al., 2011; Li et al., 2013; Cao et al., 2014; Peng et al., 2017). Therefore, it has a high potential for Zn and Cd phytoextraction in the field. However, the molecular mechanisms underlying Cd hyperaccumulation in these species have not been thoroughly investigated because of lacking genetic transformation of Cd hyperaccumulator species, which is crucial to an improved understanding of the genes important in metal hypertolerance and hyperaccumulation (Verbruggen et al., 2013). *Arabidopsis halleri* (Brassicaceae) is the only Zn/Cd hyperaccumulator reported currently accessible to genetic transformation (Hanikenne et al., 2008; Deinlein et al., 2012; Stein et al., 2017), whereas it accumulates a lower concentration of Cd in its shoots than its roots (Bert et al., 2003). To our knowledge, the only reported *Sedum* species accessible to genetic transformation is *S. erythrostictum* (Yoon et al., 2002). Therefore, the development of genetic transformation protocols for dissecting the underlying molecular mechanism in these hyperaccumulators is necessary. In this work, we successfully developed an efficient method for genetic transformation of *Sedum* species (Crassulaceae), including the Cd hyperaccumulator *S. plumbizincicola* and the non-hyperaccumulating ecotype of *S. alfredii*.

Choosing a good target tissue is critical for stable plant transformation (Karami et al., 2009). Callus induction and plant regeneration are important steps in the procedure; however, callus can be genetically unstable and regeneration from callus may result in somaclonal variation (D’Amato, 1977; Skirvin, 1978; Larkin and Scowcroft, 1981). Here, multiple shoot buds were induced from stem nodes of *S. plumbizincicola* and *S. alfredii*, and the shoot primordia were used as direct targets for *Agrobacterium* infection, resulting in efficient transformation. Accordingly, we have established a reliable method for genetic transformation of these *Sedum* species and this method may be used to facilitate a functional analysis of candidate genes involved in the molecular mechanisms underlying Cd hyperaccumulation.

## Materials and Methods

### Plant Materials and Optimization of Conditions for Shoot Induction

Stems from the non-hyperaccumulating ecotype of *S. alfredii* and the Cd hyperaccumulator *S. plumbizincicola* were used as explants for shoot induction. Freshly collected stems were first washed with water and then with 75% (v/v) ethanol for 30 s; they were then washed with sterile water three times. Subsequently, the stems were surface sterilized with 0.1% (w/v) mercuric chloride (HgCl_2_) for 6 min with gentle agitation and then washed at least four times with sterile water. The sterilized stems were cut into segments and nodal segments were selected as explants for shoot induction. The stem nodes were cultured for 3 weeks on Murashige and Skoog (MS) basal medium (pH 5.8) supplemented with 3% (w/v) sucrose and 0.9% (w/v) agar with different concentrations and combinations of phytohormones (**Table 1**). The explants were sub-cultured every week by aseptically transferring stem nodes to fresh media. Tissue was regenerated from explants by direct organogenesis and the numbers of regenerated shoot buds per node were recorded. The culture conditions were maintained at 25°C with a 10-h light (125 μmol⋅m^-2^⋅s^-1^)/14-h dark photoperiod. The medium that induced shoots most efficiently was used for the next regeneration. Regenerated shoots were then used for testing explant sensitivity to selective agents (hygromycin B and kanamycin).

**Table 1:** Effects of plant growth regulators on multiple shoot induction.

6-BA (mg/L)	NAA (mg/L)	Mean No. of shoots per node
0.1	0.1	1.30 ± 0.70ˆf
0.1	0.5	2.11 ± 0.17ˆcde
0.1	1.0	4.40 ± 0.74ˆa
0.1	1.5	1.74 ± 0.33ˆde
0.5	0.1	1.36 ± 0.31ˆe
0.5	0.5	2.50 ± 0.29ˆbcd
0.5	1.0	2.83 ± 0.07ˆbc
0.5	1.5	2.37 ± 0.27ˆbcde
1.0	0.1	3.37 ± 0.70ˆab
1.0	0.5	3.06 ± 1.12ˆbc
1.0	1.0	2.43 ± 1.08ˆbcd
1.0	1.5	3.41 ± 0.57ˆab

*The results were obtained 3 weeks after culture. Different letters indicate that mean numbers of shoots are significantly different at the 0.05 probability level.*

### Sensitivity of Shoot Buds to Antibiotics

The antibiotics hygromycin B (catalog number 10843555001, Roche) and kanamycin (catalog number KA0408, Real-times) were tested for toxicity to shoot bud growth and thereby assessed for their suitability as selective agents. To determine the optimum concentration of each, multiple shoot clusters induced from stem node explants were cultured on a shoot induction medium (MS mediumplus 0.1 mg/L 6-BA, 1.0 mg/L 1-NAA) (6-BA, catalog number B3408, Sigma–Aldrich; 1-NAA, catalog number N0640, Sigma–Aldrich) supplemented with various concentrations of hygromycin B (5, 10, 20, 30, and 40 mg/L) or kanamycin (50, 100, 150, 200, and 250 mg/L). Three replicate experiments were performed with at least nine explants cultured for each treatment. The explants were transferred to fresh medium containing the same concentration of antibiotic every week to maintain the appropriate selective pressure. The percentage of surviving explants and optimal antibiotic concentration were evaluated after 3 weeks.

### *Agrobacterium tumefaciens* Inoculation and Plant Regeneration

The plant plasmid pSN1301 contains the β-glucuronidase (GUS) reporter gene *gusA* under the control of the CaMV 35S promoter (**Supplementary Figure S1A**), while pMDC139-GFP contains an enhanced green fluorescent protein (eGFP) reporter gene *GFP* driven by double enhanced CaMV 35S promoters (**Supplementary Figure S1B**).

The *A. tumefaciens* strain C58 harboring plasmid pSN1301 or pMDC32-GFP was cultured in 5 ml liquid LB medium containing 100 mg/L kanamycin and 50 mg/L rifampicin. After shaking overnight at 200 rpm and 28°C, 2 mL of this suspension was used to inoculate 40 ml LB medium (containing the same antibiotics) and grown under the same conditions until the O.D._600_ reached 0.5–1.0. The bacterial culture was centrifuged at 4000 *g* for 10 min and the pellet was resuspended in 5 ml liquid MS medium supplemented with 100 μM acetosyringone (AS, catalog number D134406 Sigma–Aldrich). This bacterial suspension was used for transformation.

Clusters containing greater than 40 shoots, induced from stem node explants, were used as targets for *Agrobacterium*-mediated transformation. These multiple-shoot clusters were cut into small pieces, immersed in a suspension of *Agrobacteria* for 30 min, and mixed manually every 2–3 min at room temperature. The clusters were then blotted dry using sterile filter paper to remove excess *Agrobacteria* and cultivated for 3 days at 25°C on MS medium (MS medium plus 0.1 mg/L 6-BA, 1.0 mg/L 2,4-D, and 100 μM AS), in the absence of light.

After 3 days, the explants were washed three to four times using sterile water supplemented with 300 mg/L cefotaxime. They were blotted dry on sterile filter paper and then transferred to selection medium (MS medium plus 0.1 mg/L 6-BA, 1.0 mg/L NAA, 20 mg/L hygromycin and 600 mg/L cefotaxime). The culture conditions were maintained at 25°C under a 10-h light/14-h dark photoperiod. The multiple-shoot explants were transferred to fresh selective medium every week to maintain appropriate selective pressure and prevent the growth of *Agrobacteria*. Hygromycin resistant shoots that reached approximately 1 cm in length were removed and transferred to rooting medium (MS medium plus 30 mg/L hygromycin and 600 mg/L cefotaxime). *GusA* gene expression was assessed in plants that had regenerated roots. Plants with regenerated roots were transferred from selective medium to plastic pots containing soil, vermiculite, and perlite (2:1:1).

Shoots were cut from transformed plants and cultured hydroponically in 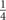 MS salt solution for rooting. The shoots began to produce roots approximately 1 week later. After a further 2–3 weeks, well-rooted plantlets were transferred to soil.

### Histochemical GUS Assay

Histochemical GUS assays were conducted as described previously (Jefferson et al., 1987). Tissue was cut from putative transformants, or alternatively wild-type plants to use as negative controls, and incubated for 4–6 h at 37°C in GUS assay buffer (0.5 mM potassium ferri- and ferrocyanide, 100 mM sodium phosphate buffer (pH 7.0), 10 mM EDTA (pH 8.0), and 0.1% (v/v) Triton X-100) containing 1 mg/mL *X*-Gluc (catalog number B5285, Sigma–Aldrich). After this incubation, green tissue was repeatedly immersed in 95% ethanol and boiled to remove chlorophyll. GUS stained tissue was visualized using a Wild M8 Stereozoom microscope (Wild Heerbrugg, Switzerland) and images were recorded using the digital camera RemoteCapture DC system (Canon Inc., Tokyo, Japan). *GusA* gene expression was assessed in transformed explants after 6 weeks of selection and an additional 3-month culture period, while expression of stable, genome-integrated reporter genes was assessed in transformed plants after rapid propagation.

### Visualization of GFP Fluorescence

Leaves and roots from putative transgenic and wild-type (negative control) plants were examined under a laser scanning confocal microscope (Zeiss LSM 510 meta; Carl Zeiss, Oberkochen, Germany) equipped with a 500–550 nm barrier filter to detect eGFP and 650–710 nm barrier filter to detect chlorophyll autofluorescence. The presence or absence of green fluorescence was compared in control and putative transgenic plants using the LSM 5 series Image Browser software (Carl Zeiss).

### Molecular Identification of Transgenic Plants

Plant genomic DNA for the polymerase chain reaction (PCR) was isolated from leaves of putative transgenic, or wild-type control, plants using DNA extraction buffer containing urea (7 M Urea, 50 M Tris-HCl [pH8.0], 500 mM NaCl, 2% SDS) (Liu et al., 2012). The specific primers used for the *gusA* gene were: forward 5′-GGTGGGAAAGCGCGTTACAAG-3′ and reverse 5′-CGGTGATACATATCCAGCCAT-3′. The PCR reaction was performed under the following conditions: 2 min at 95°C, followed by 35 cycles of 30 s at 94°C, 30 s at 55°C, and 1 min 40 s at 72°C, and a final 10-min extension period at 72°C. The reaction products were analyzed by electrophoresis on a 1% (w/v) agarose gel and visualized using a W/LMS-26E transilluminator (Gel DOC-It^TM^ 300; UVP Ltd, Upland, CA, United States).

Transgene copy number was estimated by real-time PCR (Bubner and Baldwin, 2004; Alvarez and Ordas, 2013) using the quantitation standard curve method (Ueno et al., 2011). Real-time PCR was performed using the SYBR Premix EX Taq GC Kit (Takara Bio Inc., Kusatsu, Japan) and the ABI StepOne Plus System (Applied Biosystems, Foster City, CA, United States). The primers used for real-time PCR identification of the *gusA* gene were 5′-TGTAATGTTCTGCGACGCTCAC-3′ and 5′- CTTTTTCCAGTACCTTCTCTGCCG-3′. A 50-μg portion of the genomic DNA was used as a template. To generate standard curves and quantify *gusA* copy number, serial dilutions of plasmid pSN1301 (from copy number: 10–10^6^) were made with wild-type genomic DNA and subjected to real-time PCR. The *C*_T_-values for each sample were converted to copy numbers using the standard curves.

Total RNA was isolated from leaves using the Plant RNA Purification Reagent Kit (Invitrogen, Carlsbad, CA, United States). After treatment with DNase I (Sigma–Aldrich, St. Louis, MO, United States), total RNA was reverse transcribed using the M-MLKV Reverse Transcript Kit (Promega, Madison, WI, United States) according to the manufacturer’s instructions. G*usA* gene expression was quantified by real-time RT-PCR using the comparative *C*t method (Livak and Schmittgen, 2001).

### Statistical Analysis

For all experiments, data were analyzed by a one-way analysis of variance (ANOVA) using the LSD multiple range test, and differences were considered significant at a level of 5%. IBM SPSS Statistics (ver. 19; IBM, Armonk, NY, United States) was used for the analyses.

## Results and Discussion

### Induction of Multiple Shoot Buds As Targets for *Agrobacterium* Infection

**FIGURE 1:**
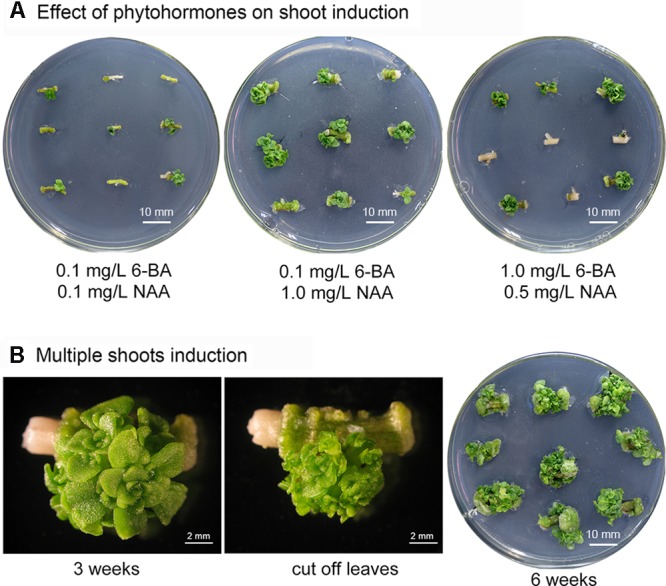
Induction of multiple *Sedum alfredii* shoots. **(A)** Stem explants grown on medium containing various concentrations of 6-BA and NAA. **(B)** Multiple shoots induced on medium containing 0.1 mg/L 6-BA and 1.0 mg/L NAA.

Phytohormones are crucial factors in plant organogenesis and development. The combination of cytokinin 6-benzyladenine (6-BA) and auxin 1-naphthaleneacetic acid (NAA) is highly effective in shoot induction and organogenesis in various Crassulaceae species including: *Hylotelephium sieboldii* (Nakano et al., 2005), *Rhodiola fastigiata* (Liu et al., 2006), *Kalanchoe blossfeldiana* (Smith and Nightingale, 1979; Castelblanque et al., 2010), and *Sempervivum tectorum* (Dobos et al., 1994). Shoot buds can be induced from stem internode-derived callus in *S. alfredii* using shoot-induction medium containing 6-BA and NAA (Zhao et al., 2009). In our preliminary trials, axillary buds were generated from *S. alfredii* stem nodes using a shoot bud induction medium containing these two phytohormones. To optimize conditions for producing multiple shoots in *S. alfredii* and to evaluate the effect of combining different concentrations of phytohormones on the number of shoots regenerated, stem node explants were cultured on MS media containing various concentrations of 6-BA (0.1, 0.5, and 1 mg/L) and NAA (0.1, 0.5, 1, and 1.5 mg/L). The stem explants were sub-cultured weekly and adventitious shoot formation was observed at leaf axils (**Figure 1A**). Medium containing 0.1 mg/L 6-BA and 1.0 mg/L NAA produced the maximum number of shoots (on average 4.40 shoots per stem node explant after 3 weeks cultivation – **Figure 1A** and **Table 1**), while fewer shoots were observed on media with low concentrations of 6-BA and NAA (**Table 1**). Concentrations of 6-BA approaching 1 mg/L were harmful and some explants died; however, surviving explants were able to generate more shoots, especially when higher concentrations of NAA were present in the medium (**Figure 1A** and **Table 1**).

After the initial 3-week shoot induction period, stem node explants with more than three shoots were removed and sub-cultured on MS medium containing 0.1 mg/L 6-BA and 1.0 mg/L NAA (**Figure 1B**). Multiple shoots began to proliferate. An average of greater than 40 shoots per stem node were observed after an additional three to 5 weeks of culture and the explants formed clusters of shoot apices at each node (**Figure 1B**).

Multiple shoots were also induced in *S. plumbizincicola* stem node explants (**Supplementary Figure S2**). Small shoots emerged from the leaf axils after a 2-week induction period on MS medium containing 0.1 mg/L 6-BA and 1.0 mg/L NAA, and several shoot buds regenerated from explants after 4 weeks of weekly sub-culture. Multiple shoot buds appeared on each *S. plumbizincicola* stem node after 10 weeks (**Supplementary Figure S2**), although *S. alfredii* shoot buds developed in a shorter time. The shoot buds were used as targets for *Agrobacterium* infection.

### Sensitivity of Shoot Bud Explants to Selective Agents

The use of a suitable selective agent at an appropriate concentration is very important in developing an efficient genetic transformation method. The particular selective agent chosen should depend on the plant, as species differ in their sensitivity to selective agents. Hygromycin and kanamycin are the most frequently used antibiotics for generating transgenic plants (An et al., 2014; Nyaboga et al., 2014). The products of selective marker genes can detoxify antibiotics, and as a result transformed plant cells harboring selective marker genes can grow and develop normally in the presence of antibiotics.

**FIGURE 2:**
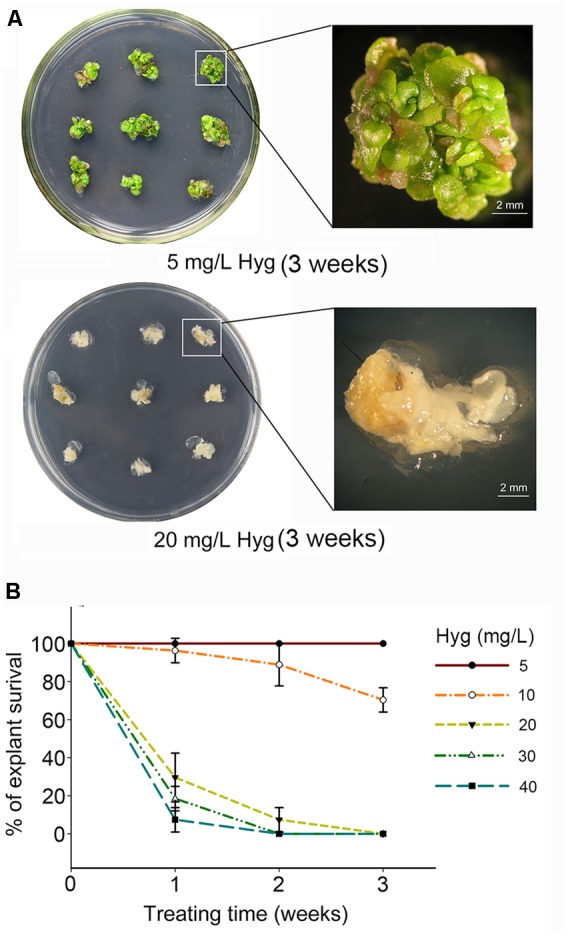
*Sedum alfredii* sensitive to hygromycin. **(A)** Growth of explants after 3 weeks on media containing different concentrations of hygromycin (Hyg). **(B)** Effect of hygromycin concentration and treatment duration on the survival of multiple-shoot explants.

The multiple-shoot clusters induced from stem node explants of *S. alfredii* were cultured on MS media containing a concentration gradient of kanamycin (50–250 mg/L) or hygromycin (5–40 mg/L). To maintain the potency of each antibiotic, the shoot clusters were sub-cultured onto fresh selection media (with the same antibiotic concentration) every week. The *S. alfredii* shoot tissue had a high tolerance to kanamycin and growth was not inhibited even at a concentration of 250 mg/L (**Supplementary Figure S3A**). However, *S. alfredii* tissue was very sensitive to hygromycin in comparison to kanamycin (**Supplementary Figure S3B**). The survival of explants declined drastically as the hygromycin concentration increased and treatment time was prolonged (**Figure 2** and **Supplementary Figure S3B**). The growth of multiple shoots was only slightly affected when the concentration of hygromycin was less than 10 mg/L (**Figure 2** and **Supplementary Figure S3B**). At a concentration of 20 mg/L hygromycin, the survival of explants was 29.63% after 1 week, 7.41% after 2 weeks, and 0% after 3 weeks (**Figures 2A,B**). After 2 weeks of culture in medium containing 30 or 40 mg/l, no shoots survived at all, indicating that excessive selection pressure had been applied (**Figure 2B**).

An appropriate concentration of selective agent is crucial for plant transformation because a suboptimal dose will result in a high frequency of escapes, while an excessive dose will not only kill untransformed tissues, but also inhibit the growth of transformed cells (Datta et al., 1990; Wilmink and Dons, 1993; An et al., 2014). Optimizing the dose of selective agent involves identifying the minimal concentration necessary to prevent regeneration or survival of untransformed tissues, while permitting the development of transformed cells (Song et al., 2012; Nyaboga et al., 2014). Our results indicated that the optimal concentration of hygromycin here was 20 mg/L and that this was suitable for the selection of transformants from an early stage. These selective conditions were also suitable for *S. plumbizincicola*. Most untransformed *S. plumbizincicola* explants turned white and died after 2 weeks on 20 mg/L hygromycin selective media (**Supplementary Figure S2**) and no surviving tissue was observed after an additional week of selection.

### High-Efficiency Transformation of *Sedum* Species and Regeneration of Transgenic Plants

**FIGURE 3:**
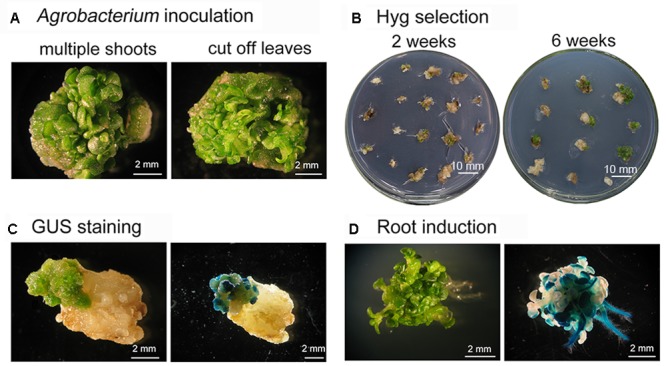
Transformation, selection, and regeneration of *Sedum alfredii* using multiple-shoot explants. **(A)** Explants with multiple shoots were isolated and infected with *Agrobacterium* for 30 min. **(B)** Selection of explants on hygromycin. Transformed explants remained green and continued to grow on selective shoot induction medium. **(C)** Regenerated tissue stained for GUS. **(D)** Root induction. Transgenic shoots rooted in MS medium containing 30 mg/L hygromycin.

A variety of factors affect the success of *Agrobacterium*-mediated genetic transformation. These include: the plant genotype, the condition of the explants, the duration of exposure to *Agrobacteria*, and the tissue culture conditions (An et al., 2014; Nyaboga et al., 2014). Among these factors, having healthy explants is critical for improving the efficiency of T-DNA insertion into genomic DNA and regenerating transformants (Wang et al., 2011). Both *gusA* and *GFP* have been widely used as transgenic reporter genes to confirm foreign genes are expressed in transformed plants because detecting their expression is easy and convenient (Jefferson et al., 1987; Prasher et al., 1992). In this study, the clusters of shoot buds induced from *S. alfredii* or *S. plumbizincicola* stem nodes were used as targets for *Agrobacterium*-mediated transformation (**Figure 3A** and **Supplementary Figure S1**). Leaves and elongated shoots were removed from explants with multiple buds and shoot primordia, and the explants were then infected with *Agrobacterium* strains harboring plasmid pSN1301 or pMDC139-GFP for 30 min (**Figure 3A** and **Supplementary Figure S1**), before being cultivated on MS medium supplemented with 0.5 mg/L 6-BA, 1.5 mg/L 2,4-D, and 100 mg/L AS, in the absence of light. After 3 days, the explants were allowed to recover for 2 weeks on shoot induction medium containing 20 mg/L hygromycin to permit the growth of transformed cells and 300 mg/L cefotaxime to suppress the growth of *Agrobacteria*. Non-transformed explants turned white or dark brown, while transformed explants remained green (**Figure 3B**). Because using a low concentration of selective agent early in the recovery of transformed cells is reportedly beneficial (Burgos and Alburquerque, 2003; Bull et al., 2009; Nyaboga et al., 2014), transformants were first cultured in shoot regeneration medium containing 20 mg/L hygromycin for 3–4 weeks and then transferred to medium supplemented with 30 mg/L hygromycin for an additional 3 weeks to effectively eliminate non-transformed cells. This method of selection has previously been effective in transforming other plant species (Sujatha and Sailaja, 2005; Fan et al., 2008; Milojevic et al., 2012). Here, shoots regenerated from the green protuberances on transformed tissue after 6 weeks of selection on the hygromycin-containing shoot induction medium (**Figure 3B**). At this point, histochemical analysis for GUS expression was performed and strong GUS staining was visible in the shoot buds and green tissue, but not in pale yellow tissue (**Figure 3C**). Well-developed hygromycin-resistant shoots were then transferred to phytohormone-free MS medium containing 20 mg/L hygromycin to induce root regeneration (**Figure 3D**). As expected, only transformed shoots survived and produced roots, while false-positive shoots failed to regenerate roots and died. To produce plantlets suitable for potting, transgenic plants were sub-cultured on rooting medium for several weeks and plantlets were then transplanted into small plastic pots.

Transformation efficiency was assessed by hygromycin resistance and GUS staining using multiple buds and shoot primordia induced from stem nodes of *Agrobacterium*-incubated explants. On average, 57.67% of the *S. alfredii* explants survived and regenerated at least one shoot after selection. Of the regenerated shoots, 82.35% expressed GUS. However, parallel experiments using *S. plumbizincicola* explants produced efficiencies of 10% hygromycin resistance and approximately 75% of shoots with observable GUS staining. The large difference in transformation efficiencies between these two species may be due to shoot organogenesis being easier in *S. alfredii* than in *S. plumbizincicola*.

### Stable Expression of the Transgene in Rapidly Propagated Transformants

**FIGURE 4:**
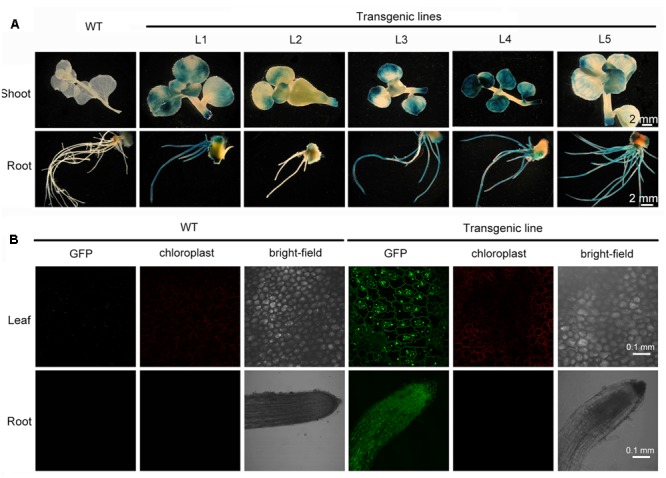
Stable expression of transgenes in rapidly propagated transformants. **(A)** Stable expression of the *gusA* gene in WT and different transgenic lines. **(B)** Stable expression of the *GFP* gene in leaf and root from WT and transgenic line.

*Sedum alfredii* and *S. plumbizincicola* can easily be made to reproduce asexually or by vegetative propagation. Shoots of putative transgenic plants were removed and cultured for rooting in hydroponic medium containing 1/4 MS basal salts for 1 week. After vegetative propagation, well-rooted plants were randomly selected and transformation was verified by GUS staining analysis. All five of the selected lines stained intensely blue in leaf, stem, and root tissue. Conversely, no blue coloration was observed in wild-type plants (**Figure 4A**), indicating that the *gusA* gene had stably integrated into the transformed plants’ genome and was functional. The intensity of GUS staining varied among the lines (**Figure 4A**) possibly due to the different locations or copy numbers of the transgene influencing its expression level in the transformants. Another reporter gene, *GFP*, was introduced by *Agrobacterium*-mediated transformation into *S. alfredii* explants. GFP expression was observed in leaf and root cells of transgenic plants (**Figure 4B**), whereas no GFP fluorescence was detected in non-transformed wild-type plants (**Figure 4B**).

### Molecular Identification of Transgenic Plants

**FIGURE 5:**
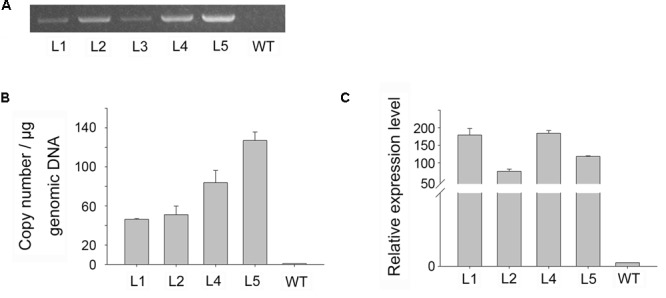
Molecular analysis of transgenic plants. **(A)** PCR analysis of genomic DNA for the *gusA* gene in putative transgenic, and WT, plants. **(B,C)** Real-time PCR analysis of genomic DNA for *gusA* gene copy number **(B)** and of cDNA for *gusA* gene expression level **(C)**.

To confirm that foreign genes had been inserted into the transgenic plants, genomic DNA was isolated from GUS positive and wild-type plants, and a PCR was performed using gene-specific primers. PCR generated the predicted amplicon (1504 bp) for the *gusA* gene in transformed plants, but no amplicon in wild-type plants (**Figure 5A**), confirming the presence of the *gusA* gene in transgenic plants.

Although multiple copies of a transgene may be useful for overexpression experiments, single-copy transformation is preferred for most applications due to its stability (Meyer, 1998). Song et al. (2012) demonstrated that transgenic plants with high transgene copy numbers could be distinguished from those with low copy numbers using real-time PCR (Bubner and Baldwin, 2004; Ueno et al., 2011; Alvarez and Ordas, 2013). Four independent *gusA*-positive (PCR) lines were analyzed using quantitative real-time PCR to estimate the transgene copy number of *gusA* in genomic DNA samples. We normalized the data using the cycle threshold (*C*_T_) value of *gusA*, which occurs as a single gene copy in the SN1301 plasmid. Using these standards, the *gusA* copy number proved to be highly variable in the transgenic plants, but provided values that were approximately multiples (i.e., 1:1:2:3) among the different lines, ranging from 46.33 to 126.98 copies per microgram of genomic DNA (**Figure 5B**).

The transgenic lines were analyzed by real-time RT-PCR to verify the expression of the *gusA* gene at the RNA level. The *gusA* gene was expressed in all four transformants, and the expression levels showed significant differences between the lines, ranging from 75.77 (line 2) to 184.08 (line 4) (**Figure 5C**). No RNA from the *gusA* gene was detected in the non-transformed control plants. The real-time PCR transcript data ruled out residual Agrobacteria activity and confirmed true transformation events. These results demonstrated that the target gene had successfully integrated into the plant genome and was stably expressed in transgenic plants, but no significant correlations were found between transgene copy number and GUS activity levels.

## Conclusion

By inducing multiple shoots from stem nodes and using the shoot apices as targets for *Agrobacterium* infection, we successfully established an efficient method of genetic transformation for the Cd/Zn hyperaccumulator *S. plumbizincicola* and the non-hyperaccumulating ecotype of *S. alfredii*. This callus-free protocol is a simple, highly efficient approach to genetic transformation in species of *Sedum*. By facilitating genetic manipulation of these plants this transformation method may allow the molecular mechanisms underlying Cd hyperaccumulation to be investigated by RNA interference, CRISPR/Cas9, and gene overexpression. Furthermore, because species of the *Sedum* genus are crassulacean acid metabolism (CAM) plants, this transformation method may also facilitate investigation into the molecular mechanisms regulating the temporal CO_2_ pump and perhaps assist in introducing CAM into C_3_ crops, improving the efficiency of plant water-use (Borland et al., 2014).

## Author Contributions

WX conceived the original screening and research plans. HL performed most of the experiments. HZ provided technical assistance to HL. WX supervised the experiment. HL and WX designed the experiments and analyzed the data. HL, HZ, and WX conceived the project and wrote the article with contributions of all the authors. LW and WX supervised and complemented the writing.

## Conflict of Interest Statement

The authors declare that the research was conducted in the absence of any commercial or financial relationships that could be construed as a potential conflict of interest.

## Supplementary Material

FIGURE S1 Schematic representation of the T-DNA region. **(A)** pSN1301. **(B)** pMDC139-GFP.

FIGURE S2 Transformation of *Sedum plumbizincicola*. **(A)** Induction of multiple shoots. **(B)** Selection of explants using hygromycin. **(C)** Root induction on selective medium without phytohormones. **(D)** GUS staining of putative transgenic plants.

FIGURE S3 Testing *Sedum alfredii* for sensitivity to kanamycin and hygromycin. **(A)** The effect of varying kanamycin concentration on the growth of explants. **(B)** The effect of varying hygromycin concentration on the growth of explants.
