# Supplementary figures and images for: A Genetic Transformation Method for Cadmium Hyperaccumulator Sedum plumbizincicola and Non-hyperaccumulating Ecotype of Sedum alfredii
Source: Front Plant Sci. 2017 Jun 16;8:1047. doi: 10.3389/fpls.2017.01047 (PMC5472854; doi:10.3389/fpls.2017.01047)

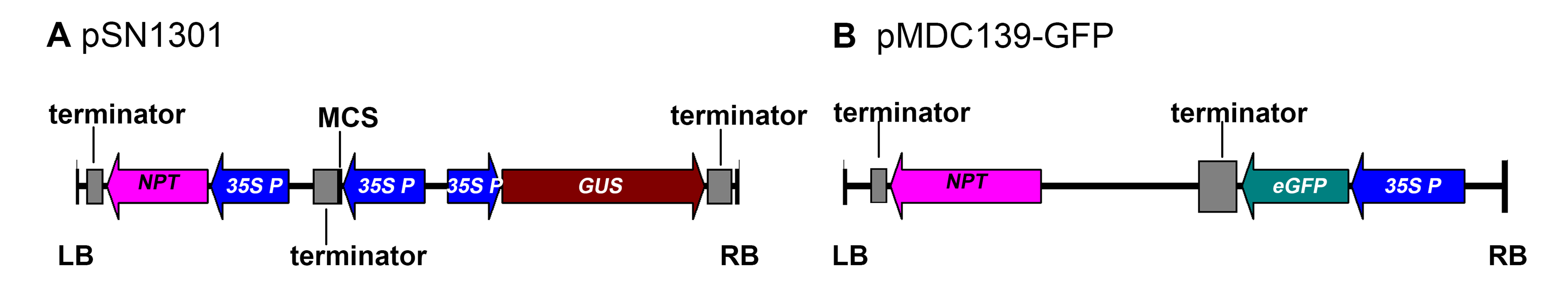

Supplement: FIGURE S1 — Schematic representation of the T-DNA region. (A) pSN1301. (B) pMDC139-GFP. [file Image_1.TIF]

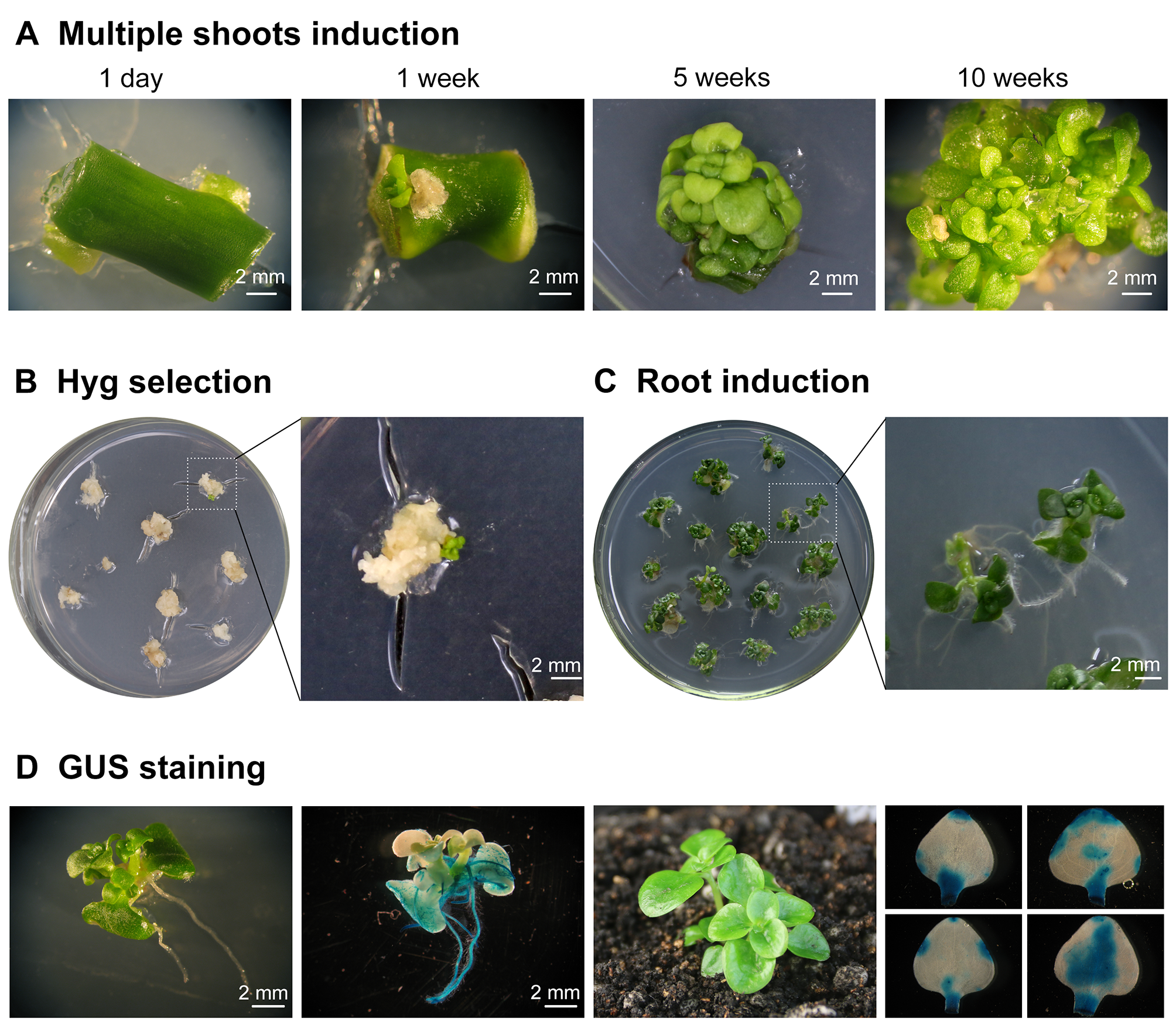

Supplement: FIGURE S2 — Transformation of Sedum plumbizincicola. (A) Induction of multiple shoots. (B) Selection of explants using hygromycin. (C) Root induction on selective medium without phytohormones. (D) GUS staining of putative transgenic plants. [file Image_2.TIF]

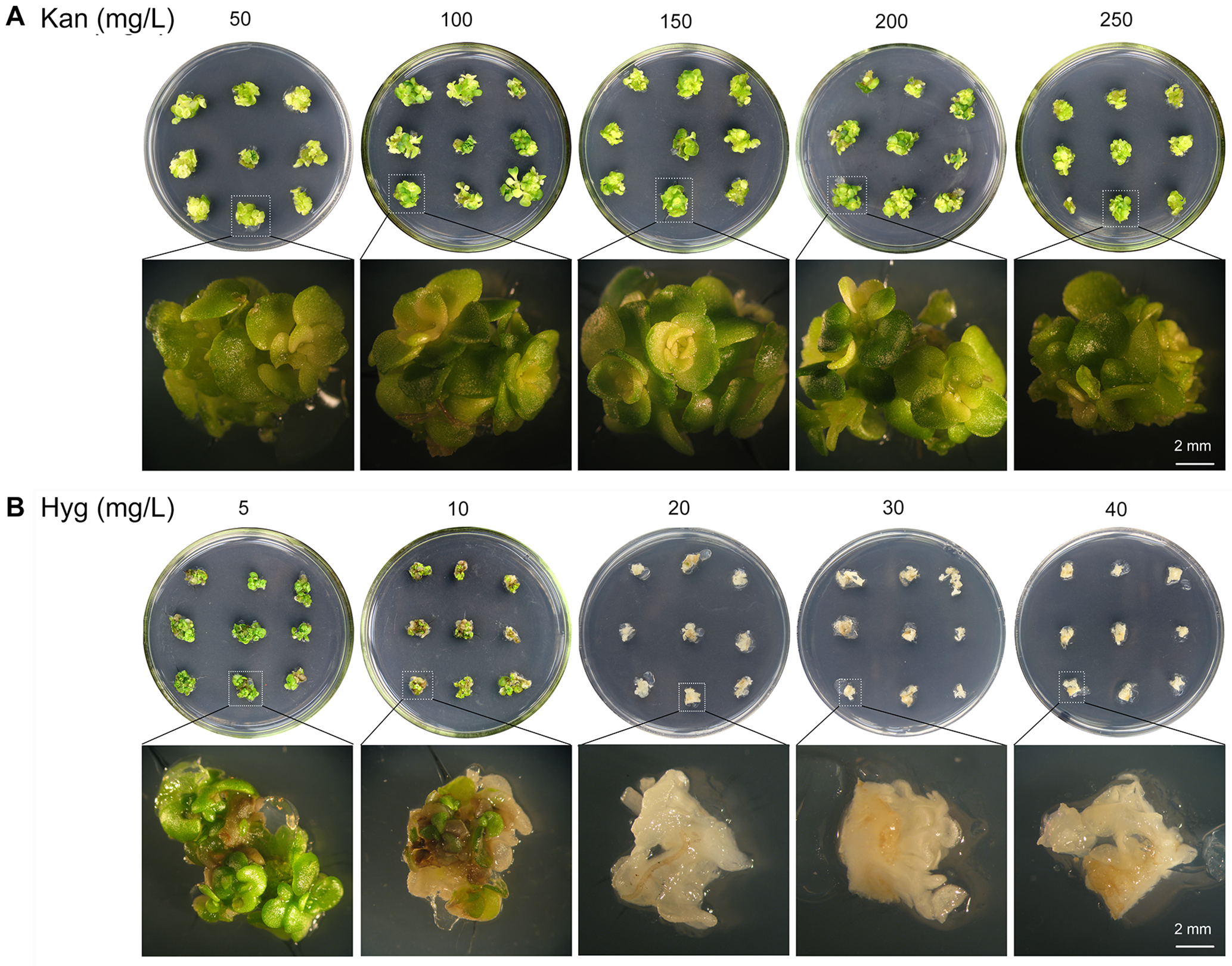

Supplement: FIGURE S3 — Testing Sedum alfredii for sensitivity to kanamycin and hygromycin. (A) The effect of varying kanamycin concentration on the growth of explants. (B) The effect of varying hygromycin concentration on the growth of explants. [file Image_3.TIF]
